# Hypothetico‐Deductive Reasoning in Direct Oral Anticoagulant Trial Meta‐Analyses: A Meta‐Epidemiological Study

**DOI:** 10.1111/fcp.70118

**Published:** 2026-11-13

**Authors:** Quentin Remy, Arthur Gougeon, Paul Riallin, Rémy Boussageon, Jean‐Christophe Lega

**Affiliations:** ^1^ Département de médecine générale Université Claude Bernard Lyon 1 Lyon France; ^2^ Laboratoire de Biométrie et Biologie Evolutive UMR 5558, CNRS Université Lyon 1, Université de Lyon Villeurbanne France; ^3^ Service de Rhumatologie, Hôpital Lyon Sud Hospices Civils de Lyon Lyon France; ^4^ Service Hospitalo‐Universitaire de Pharmacotoxicologie, Pôle Santé Publique Hospices Civils de Lyon Lyon France

**Keywords:** direct oral anticoagulants, false positives, hypothetico‐deductive reasoning, meta‐analysis, meta‐epidemiology, type 1 error

## Abstract

**Background:**

Direct oral anticoagulants (DOAC) have become standard therapy for thrombosis prevention since 2008. Meta‐analyses (MAs) of randomized controlled trials (RCT) are considered high‐level evidence supporting clinical guidelines. However, methodological biases may yield false‐positive findings, particularly through inadequate hypothesis formulation, multiple testing without alpha‐risk correction, and selective outcome reporting. No study has systematically assessed the risk of false positives in MA evaluating DOAC through hypothetico‐deductive reasoning.

**Objective:**

This study aimed to assess methodological features relevant to confirmatory inference in MA of RCTs evaluating DOACs, including hypothesis specification and multiplicity management, and to quantify nominal exposure to type I error under specified assumptions.

**Methods:**

Following a pre‐specified protocol, we conducted a meta‐epidemiological study using embase and PubMed databases. We included MA of RCT evaluating four EMA‐approved DOAC. Two independent authors performed study selection. For each MA, we calculated the nominal family‐wise probability of at least one type I error under the global null hypothesis, assuming independent tests. We assessed the presence of directional hypotheses, primary outcome definition, pre‐specified protocols, sensitivity analyses, and spin in conclusions.

**Results:**

Ninety‐three MA were included (mean 34 118 patients; seven RCT per MA). The median nominal family‐wise type I error probability was 40%, and only 2.2% of MAs reported alpha‐risk management, 26.9% had pre‐specified protocols, and 14.0% reported directional hypotheses. Only 5.4% combined a clearly specified directional hypothesis with a single primary objective and well‐defined outcome. Spin was present in 30.1% of conclusions.

**Conclusion:**

Most MAs showed substantial uncorrected nominal exposure to multiplicity; this estimate is a theoretical indicator and not the probability that a published conclusion is false. Many MAs also lacked explicit hypotheses and frequently exhibited spin. Strengthening reliability requires integrating genuine hypothetico‐deductive approaches and adapting methodological guidelines.

## 1 Introduction

The prevention and treatment of thromboembolic diseases have long relied primarily on vitamin K antagonists (VKA), particularly warfarin, which had been considered the reference treatment for such conditions. Since their marketing authorization in 2008, direct oral anticoagulations (DOAC) have assumed a prominent role in the prevention and management of thrombosis, especially in non‐valvular atrial fibrillation, venous thromboembolism, and to a lesser extent in postoperative thromboprophylaxis following total hip or knee replacement surgery [1, 2, 3, 4, 5, 6]. In the context of non‐valvular atrial fibrillation, DOAC has demonstrated a significant reduction in the incidence of intracranial hemorrhage [7, 8, 9, 10]. DOAC has also demonstrated non‐inferiority in the treatment of deep venous thromboembolism and pulmonary embolism, as well as in their secondary prevention, with a lower risk of major bleeding events [11, 12].

In addition to their efficacy and relative safety in the management of thrombotic disorders, DOAC present several advantages. Unlike VKA, they do not require routine coagulation monitoring and are associated with fewer drug–drug and drug–food interactions. Their benefits are therefore potentially multifaceted. Accordingly, the most recent guidelines from the European Society of Cardiology and the American College of Chest Physicians strongly support the use of DOAC in these various indications, based on data from randomized controlled trials (RCT), observational studies, and meta‐analyses (MAs) [13, 14, 15, 16, 17].

MAs of RCTs are commonly placed at the top of evidence hierarchies and frequently inform clinical guidelines. However, their inferential role differs from that of individual RCTs, and they should not be regarded as automatically providing a higher level of evidence [18, 19]. By synthesizing data from RCT, they are often used to assist clinicians in decision‐making. As such, they support clinical practice guidelines, which are easier to interpret than individual studies. However, despite these strengths, MAs are not immune to methodological biases, which may yield false‐positive findings, namely, the erroneous conclusion of a treatment effect. These biases, particularly in the case of DOAC, may stem from several sources. Trials differ markedly in patient populations, dosing regimens, assumptions of class effect, and selected endpoints and may lead to misleading conclusions through indirectness. In addition, false‐positive findings can arise from selective outcome reporting, publication bias [20], or the inclusion of high‐risk‐of‐bias trials. Finally, a hypothetico‐deductive perspective—requiring explicit distinction between exploratory and confirmatory endpoints and proper handling of multiplicity—could strengthen the appraisal of evidence robustness in DOAC trials, yet this dimension is not currently accounted for within the GRADE framework [18]. To our knowledge, no study has yet systematically addressed the risk of false positives in MAs of RCT involving DOAC, nor examined how hypothetico‐deductive reasoning might contribute to refining them [21].

This study aims to assess methodological features relevant to confirmatory inference in MA of RCTs evaluating DOACs and to quantify nominal exposure to type I error under the global null hypothesis and an independence assumption.

## 2 Methods

This work was conducted according to a pre‐specified protocol (see Appendix A) registered on June 25, 2025 (OSF platform, https://osf.io/8tjx9). We reported our study in accordance with the Preferred Reporting Items for Systematic Reviews and Meta‐Analyses (PRISMA) checklist (see Appendix C).

### 2.1 Eligibility Criteria

MAs were eligible for inclusion if they met the following criteria. The population included adult participants (aged 18 years or older) with one of the following conditions: non‐valvular atrial fibrillation, pulmonary embolism, deep vein thrombosis, total hip replacement, or total knee replacement. The intervention arm had to involve one of the four DOAC authorized in Europe—apixaban, rivaroxaban, edoxaban, or dabigatran—either assessed individually or in combination with other DOAC or antithrombotic agents, provided sufficient data were specifically available for DOAC. Comparators could consist of placebo or any antithrombotic agent, including VKA, low‐molecular‐weight heparin, unfractionated heparin, antiplatelet agents, or another DOAC, administered alone or in combination. Eligible outcomes were efficacy, safety, or both, as assessed in the MA. The study design had to be a MA of RCT.

The main exclusion criteria were as follows: MA involving pediatric populations, antiphospholipid syndrome, unapproved comparators or interventions by the European Medicines Agency (e.g., betrixaban), non‐pharmacological strategies, or DOAC withdrawn from the market (e.g., ximelagatran or darexaban). We also excluded MA that grouped multiple antithrombotic agents in such a way that individual data on DOAC could not be clearly distinguished. Lastly, we excluded MA including observational data and network MAs.

### 2.2 Search Strategy

A comprehensive literature search was conducted in early May 2025, following an initial exploratory screening phase performed from January to April 2025 to refine search terms and strategy. The databases used were Embase and PubMed, with no date restrictions. Grey literature was not included. The search strategy combined terms related to anticoagulants and their clinical indications, with filters applied for RCT and MA. Detailed search equations for each database are provided in Table S1.

### 2.3 Study Selection and Data Extraction

Two independent authors (QR, PR) conducted study selection based strictly on the inclusion and exclusion criteria outlined above. A two‐stage screening process was applied: first by title and abstract, followed by full‐text review. Disagreements were resolved by consensus among authors, with a third‐party adjudicator available if necessary. Selection and data extraction were performed using the Covidence Systematic Software [22].

### 2.4 Data Extraction

Data extraction was performed by a single author (QR). The following data were extracted and categorized into an extraction table. Descriptive variables included title, PubMed identifier, digital object identifier, first author, year of publication, journal, type of DOAC, type of comparator, clinical indication of interest, and the number of RCT and participants included in each MA. Quantitative variables included the number of inferential tests capable of supporting a clinical efficacy or safety claim and the nominal significance threshold *α*. We counted tests of primary and secondary clinical outcomes and formal interaction tests; subgroup estimates were counted only when accompanied by a distinct inferential test. Heterogeneity statistics (*I* ^2^ and Cochran's *Q*), publication‐bias tests, and descriptive analyses were excluded. Qualitative variables included the presence of a directional hypothesis, defined as an explicit expectation of a difference between treatments in the Introduction or Methods section. Hypotheses were classified as explicit, ambiguous or implicit, or absent. The primary outcome was characterized by its objective (efficacy, safety, or both) and type (undefined, multiple non‐hierarchized, single, composite, conjunctive co‐primary). We also combined those three variables (directional hypothesis, objectives, primary outcome) to assess whether the hypothesis was well predefined (clearly stated, directional, with a predefined single outcome) or not. We recorded the presence of a pre‐specified protocol, at least one positive trial within the MA, sensitivity analyses, class effect analysis, a risk of bias assessment, and any adjustment for multiple testing. As a post hoc analysis, we assessed whether each MA was explicitly described as prospective or explicitly stated that it adopted the hypothesis tested in the first published RCT included in the synthesis. We classified conclusion language as confirmatory, exploratory, or ambiguous using prespecified expressions (Table S2). This classification concerned wording only and did not imply that confirmatory inference was methodologically justified. Finally, we examined the presence of spin in the conclusion, which was defined separately as outcome‐related discordance between the primary results and the conclusion, for example, when a non‐superior primary outcome was replaced by a favorable secondary outcome in the conclusion.

For interpretation, variables were grouped into three domains: (1) confirmatory architecture, including temporal prespecification, hypothesis formulation, outcome hierarchy, and multiplicity control; (2) robustness safeguards, including risk‐of‐bias assessment, sensitivity analyses, and class‐effect assessment; and (3) reporting and interpretation, including conclusion wording and outcome‐related spin.

### 2.5 Risk of Bias

Although qualitative tools for appraising MA were available [23], the aim of this work was to evaluate the risk of false‐positive findings and the validity of conclusions in MA through the lens of hypothetico‐deductive reasoning. Therefore, no formal risk of bias tool was applied.

### 2.6 Synthesis of Results

A combined quantitative and qualitative synthesis approach was used. For each MA, we calculated the nominal family‐wise probability of at least one type I error under the global null hypothesis [24, 25], assuming *n* independent tests conducted at a per‐test significance level *α*:

*P* (any type I error|global null) = 1 − (1 − *α*)*ⁿ*.

This quantity is a theoretical indicator of exposure to multiplicity. It is not the observed false‐positive rate, the false discovery rate, or the probability that a reported significant result or conclusion is false. Because outcomes within a MA are often correlated, independence was treated as a simplifying assumption.

An analysis of the hypothesis formulation, statistical testing, nature of the primary objective, and how these elements were integrated into the MA conclusions (either confirmatory or exploratory) was performed.

## 3 Results

### 3.1 Results of the Search

The search identified 636 records. After title and abstract screening, 478 articles were excluded, leaving 146 for full‐text assessment. Of these, 53 were excluded, mainly due to missing data (short formats or conference abstracts). Finally, 93 MA met the eligibility criteria and were included (Figure 1).

**FIGURE 1:**
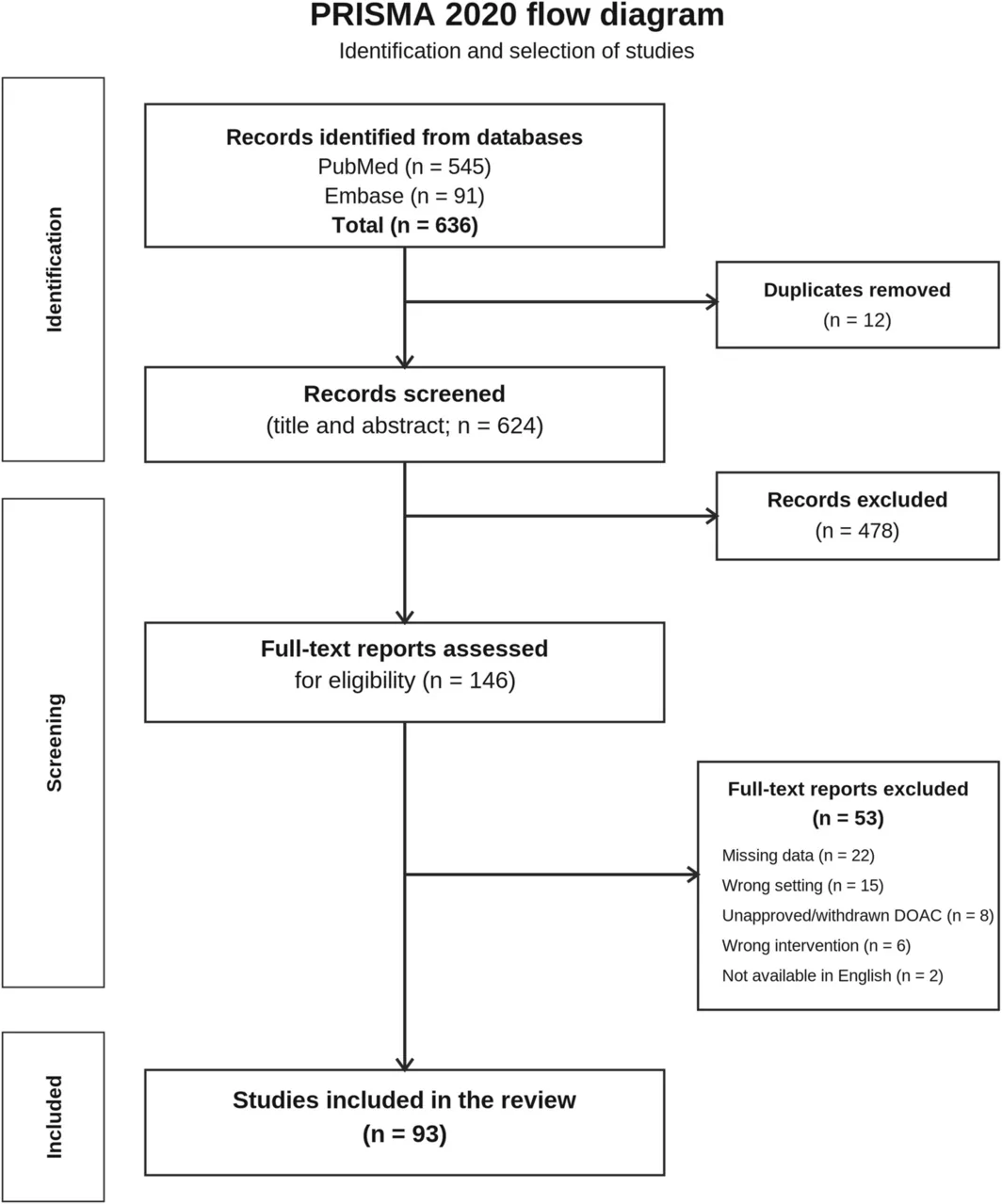
PRISMA 2020 flow diagram of study selection.

### 3.2 Characteristics of Studies

All included studies were MA of RCT, with a mean number of 34 118 patients and seven RCT per MA. Regarding publication date, most studies were published between 2008 and 2019 (*k* = 63, 67.7%). A protocol was pre‐specified for 26.99% (*k* = 25) of primary outcomes. In the remaining 73.1% of cases, the MA did not report a specific protocol but indicated adherence to the preferred reporting items for systematic reviews (PRISMA) guideline.

The largest proportion of MA (47.3%) focused on non‐valvular atrial fibrillation, 22.6% on VTE, 12.9% on total knee or hip replacement, and the remaining studies addressed one or more combined indications (see details in Table 1). The proportion of studies that assessed the risk of bias was 80.6% (*k* = 75). In terms of intervention, 47.2% of the studies evaluated all four DOAC (apixaban, rivaroxaban, dabigatran, edoxaban), 29.0% focused on three DOAC, 8.6% on two, and 15.1% on a single DOAC. The total number of MA supporting a class effect was 84.9% (*k* = 79), with their results and conclusions based on at least two DOACs. Overall, 89.2% (*k* = 83) of the MA included at least one positive study supporting the results of meta‐analytic treatment effect for each primary outcome (efficacy and/or safety).

**TABLE 1:** Characteristics of meta‐analyses.

	*k* (%)
Mean number of patients per MA	34 118
Mean number of RCT per MA	7
Year of publication	
2008–2014	20 (21.5)
2015–2019	43 (46.2)
2020–2025	30 (32.3)
Medical conditions	
NVAF	44 (47.3)
VTE	21 (22.6)
Total hip/knee replacement	12 (12.9)
NVAF and VTE	12 (12.9)
NVAF and VTE and total knee/hip replacement	4 (4.3)
MA with specific populations (*k* = 40)	
Cancers	8 (8.6)
Cardioversion	6 (6.5)
Elderly patients	6 (6.5)
Dialysis/chronic kidney disease	4 (4.3)
Obesity	3 (3.2)
Ischemic heart disease/stroke	3 (3.2)
Others^a^	10 (10.8)
Intervention	
Including one DOAC	14 (15.1)
Apixaban	5 (5.4)
Rivaroxaban	5 (5.4)
Edoxaban	1 (1.1)
Dabigatran	3 (3.2)
MA including two DOAC	8 (8.6)
Apixaban, rivaroxaban	5 (5.4)
Apixaban, edoxaban	1 (1.1)
Rivaroxaban, edoxaban	1 (1.1)
Rivaroxaban, dabigatran	1 (1.1)
MA including three DOAC	27 (29.0)
Apixaban, rivaroxaban, dabigatran	15 (16.1)
Apixaban, rivaroxaban, edoxaban	10 (10.8)
Rivaroxaban, dabigatran, edoxaban	2 (2.2)
MA including four DOAC	44 (47.3)
Apixaban, rivaroxaban, dabigatran, edoxaban	43 (46.2)
DOAC and one antiplatelet drug	1 (1.1)
Controls	
VKA	50 (53.8)
Low molecular weight heparin	17 (18.3)
VKA and low molecular weight heparin	6 (6.5)
Aspirin	4 (4.3)
Others^b^	16 (17.2)
Other specificities	
DOAC with anticoagulants	9 (9.7)
Monotherapy vs. dual antithrombotic therapy	2 (2.1)
Dual vs. triple antithrombotic therapy	2 (2.1)
Analysis DOAC and low molecular weight heparin	3 (3.2)
RCT subgroups	4 (4.3)
Reduced doses	2 (2.1)
Exposed vs. unexposed DOAC	4 (4.3)

Abbreviations: DOAC, direct oral anticoagulant; MA, meta‐analysis; NVAF, non‐valvular atrial fibrillation; RCT, randomized controlled trial; VKA, vitamin K antagonists; VTE, venous thromboembolism.

^a^ Peri procedures, diabetes, intraocular hemorrhage, and cerebral venous thrombosis.

^b^ Placebo, warfarin/placebo, VKA/low molecular weight heparin/placebo/aspirin, low‐molecular‐weight heparin/aspirin, low‐molecular‐weight heparin/VKA/placebo, aspirin/VKA/placebo, aspirin/DOAC, and triple antithrombotic therapy.

In most cases, the comparator was a VKA (53.8%), followed by low molecular weight heparin (18.3%). The remaining comparators included combinations such as VKA and low molecular weight heparin, aspirin, or other antithrombotic regimens. Forty MAs (43.0%) investigated specific populations. Finally, 8.6% of the MA included comparators that consisted of both DOAC and other anticoagulants.

### 3.3 Nominal Family‐Wise Type I Error Probability and Alpha‐Risk Management

The number of statistical tests per MA ranged between 2 and 67, with 25% and 75% quartiles at 6 and 19, and a median of 10 (Table 2). All studies applied a significance threshold of *α* = 0.05.

**TABLE 2:** Nominal family‐wise type I error probability in meta‐analyses. Probabilities were calculated as 1 − (1 − *α*)*ⁿ*, assuming a global null hypothesis, independent tests, and *α* = 0.05 for each test.

Distribution	Number of tests	Nominal family‐wise type I error probability (proportion)
Minimum	2	0.10
First quartile	6	0.26
Median	10	0.40
Third quartile	19	0.62
Maximum	67	0.97

Under the global‐null and independence assumptions, the distribution of nominal family‐wise probabilities was broad (Figure 2), with a median of 0.40. Overall, 15.1% of MA had a nominal probability below 0.20, 46.2% had probabilities between 0.20 and 0.50, and 38.8% had probabilities above 0.50. In addition, 6.5% approached unity (at least 0.8). These values indicate exposure to multiplicity and should not be interpreted as the probability that a published conclusion was false.

**FIGURE 2:**
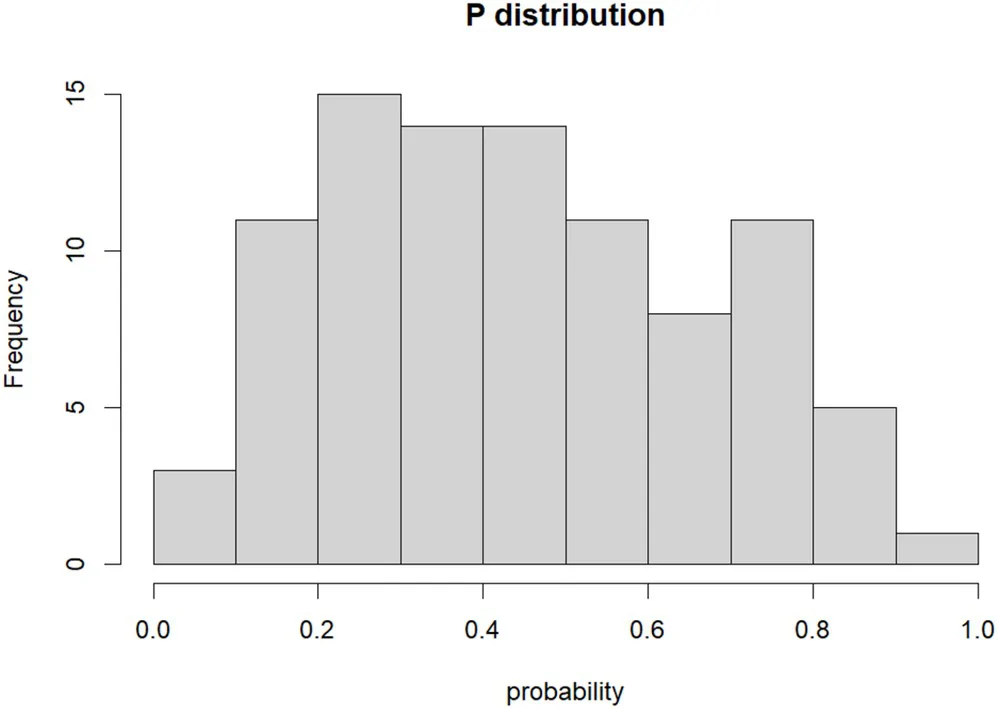
Nominal family‐wise type I error probability per meta‐analysis using: *p* = 1‐(1 − *α*)^*n*^.

Only 2.2% (*k* = 2) of the MA described any management of the alpha risk. The two methods used were Bonferroni correction and trial sequential analysis.

### 3.4 Explicit Hypothesis

Directional hypotheses (i.e., explicit expectation of a difference between drugs) were reported in 14.0% (*k* = 13) of cases, an implicit or ambiguous formulation in 25.8% (*k* = 24), and no clear hypothesis in 60.2% (*k* = 56). Primary outcome of MA was multiple objectives—typically both efficacy and safety—without any explicit hierarchical prioritization in 73.1% (*k* = 68) of cases. A single objective was specified in 26.9% (*k* = 25), including efficacy (*k* = 9), safety (*k* = 14), or a co‐primary outcome requiring both efficacy and safety to be positive (*k* = 2). Primary outcome was not clearly defined or multiple without hierarchization in 35.4% for efficacy outcomes and 34.8% for safety (Table 3).

**TABLE 3:** Type of primary outcome.

Type of outcome	Number of studies	
	Efficacy (*n* = 79), *n* (%)	Safety (*n* = 86), *n* (%)
No primary outcome reported	0	1 (1.2)
Not clearly defined or multiple non‐hierarchized	28 (35.4)	30 (34.9)
Multiple primary outcomes	0	1 (1.2)
Composite primary outcome	36 (45.6)	19 (22.1)
Conjunctive co‐primary outcomes	1 (1.3)	2 (2.3)
Single primary outcome	14 (17.7)	33 (38.4)

Finally, across all MA, 5.4% (*k* = 5) combined a clearly specified directional hypothesis with a single primary objective and a well‐defined primary outcome (whether single, composite, hierarchical, or co‐primary). None of the included MA were explicitly described as prospective syntheses, and 96.8% did not explicitly report adopting the hypothesis tested in the first published RCT included in the analysis; this information could not be determined in 3.2% (*k* = 3) of the cases.

### 3.5 Sensitivity Analysis

The proportion of MA that conducted a sensitivity analysis was 49.5% (*k* = 46). The most frequently applied approaches were leave‐one‐out analyses (17.2%, *k* = 16) and analyses based on risk of bias (12.9%, *k* = 12), whereas class effect analyses were rare (4.3%, *k* = 4). A heterogeneous set of other sensitivity analyses was reported in 24.7% of cases (*k* = 23).

### 3.6 Reporting of Results and Spin in the Conclusion

Overall, 55.9% (*k* = 52) of MAs used clearly confirmatory wording in their conclusions, 29.0% (*k* = 27) used exploratory wording, and 15.1% (*k* = 14) were ambiguous. Four MAs (4.3%) combined a clearly defined hypothesis with confirmatory wording, and only two of these based their conclusion on the primary outcome. Forty‐eight MAs (51.6%) used confirmatory wording despite a poorly defined hypothesis. No MA using exploratory wording had a clearly stated hypothesis.

Among studies focusing on efficacy, 11.8% (*k* = 11) exhibited spin in the conclusion, whereas 23.7% (*k* = 22) of those addressing safety did so. Overall, 30.1% (*k* = 28) of studies displayed at least one instance of spin related to either efficacy or safety.

## 4 Discussion

MAs of RCT are widely regarded as a high level of evidence in health technology assessment, and they often serve as a direct basis for regulatory and reimbursement decisions. In this context, it is imperative to adopt a hypothetico‐deductive rather than an exploratory approach [19]. However, only 26.7% of the included MAs specified a single primary objective, and only 5.4% combined a clearly stated directional hypothesis with a single primary objective and a well‐defined primary outcome. These characteristics alone do not establish whether the hypotheses were formulated independently of the available evidence. Moreover, MA should adopt a unique hypothesis independently of the data analysis to respect the hypothetico‐deductive logic, which is the case for formal prospective MA or those that follow the hypothesis tested in the first published RCT [26]. Thus, our post hoc analysis reveals that this is rarely extended to MA. None of the included MA were explicitly described as prospective syntheses; and in 96.8% of MA, the authors did not explicitly report adopting the hypothesis tested in the first published RCT included in the synthesis. These findings were accompanied by infrequent reporting of multiplicity control, absence of protocol registration in 73.1% of MAs, and omission of sensitivity analyses in nearly half of them. However, our study did not quantify the extent to which these methodological characteristics were associated with false‐positive findings. Most MA (84.9%) assumed a class effect, although such an assumption was not clearly supported by evidence [27].

Type I error control and statistical power address different threats to inference. Statistical results should be interpreted in light of both the nominal type I error [28, 29, 30, 31] and the power to detect a clinically relevant effect [32]. Pooling generally increases precision, but power may remain limited for rare outcomes, small effects, or heterogeneous treatment effects. For a given prior probability of a true effect and a fixed significance threshold, lower statistical power reduces the positive predictive value of a statistically significant finding without increasing the nominal type I error probability under the null hypothesis. Our analysis did not estimate outcome‐specific power or *β* risk and should therefore be interpreted only as an assessment of nominal exposure to type I error under the stated assumptions. RCTs and MAs have distinct and complementary inferential roles. A well‐conducted RCT may provide a confirmatory test of a prespecified hypothesis, whereas a MA synthesizes treatment effects across trials, increases precision when appropriate, and evaluates between‐study heterogeneity. MA should therefore not automatically be placed above individual RCTs in a linear hierarchy of evidence but should be appraised in parallel with the methodological validity and results of the component trials [33].

Furthermore, outcome‐related spin was identified in 30.1% of the MAs, indicating discrepancies between the primary results and the emphasis placed on outcomes in the conclusions. Assessing both the efficacy and safety of a drug is inherently legitimate, even essential, in the therapeutic evaluation of antithrombotic drugs. However, the absence of clearly formulated and hierarchized objectives—even when relevant outcomes are used (single, composite, or co‐primary)—increases the risk of selective reporting [34, 35, 36]. In this context, it is noteworthy that despite the frequent absence of an explicit hypothesis, more than half of the MA presented confirmatory wording conclusions. However, this linguistic classification does not establish whether the methodological requirements for confirmatory inference were satisfied. This rate is consistent with that reported in the international literature [37, 38, 39, 40]. Risk‐of‐bias assessments and sensitivity analyses should not be treated as defining components of hypothetico‐deductive reasoning. They are robustness safeguards used to determine whether conclusions depend on assumptions or study characteristics. Similarly, spin is an interpretive phenomenon that may arise from weak prespecification but also from selective emphasis, unaddressed bias, or overgeneralization. Our operational definition captured only outcome‐related discordance between primary results and conclusions.

To our knowledge, this is the first study to quantify the nominal family‐wise type I error probability in DOAC MAs, following the REB approach [21]. Among the potential implications for research and future methodological recommendations, several directions may be considered: mandating the existence of a pre‐specified protocol, adapting PRISMA guidelines [41] to explicitly require the declaration of the exploratory or confirmatory nature of a review, systematically associating this declaration with a directional hypothesis and a clearly prioritized primary outcome, considering the inclusion, within existing spin classifications [42, 43], of a specific category related to the absence or weakness of hypotheses.

Our study has several limitations. Our spin classification focused on outcome‐related discordance and did not capture all possible forms or mechanisms of spin. Data extraction was performed by a single investigator. No grey literature was used. We did not assess outcome‐specific statistical power or *β* risk. In addition, we included only conventional MA, excluding those based on individual participant data, complex subgroup analyses requiring advanced statistical modeling, network MA, and Bayesian approaches. These methodological choices limit the generalizability of our findings.

In conclusion, most MAs assessing DOAC lacked an explicit hypothesis. Under the global null hypothesis and the assumption of independent tests, the median nominal family‐wise type I error probability was 40%. The frequent absence of a pre‐specified protocol, vague outcome definitions, and spin further undermine their confirmatory value. Strengthening reliability requires integrating a genuine hypothetico‐deductive approach and adapting methodological guidelines.

## Author Contributions

Quentin Remy drafted the protocol; Paul Riallin is the second reviewer; Rémy Boussageon, Jean‐Christophe Lega, and Arthur Gougeon revised the protocol; Arthur Gougeon and Jean‐Christophe Lega supervised and designed the methodology and are the guarantors of the review.

## Funding

The authors have nothing to report.

## Ethics Statement

Due to the meta‐epidemiological nature of this work, no ethical approval was required.

## Consent

Due to the meta‐epidemiological nature of this work, no patient consent was required.

## Conflicts of Interest

The authors declare no conflicts of interest.

## Permission to Reproduce Material From Other Sources

No documents from any other source were used.

## Clinical Trial Registration

Due to the meta‐epidemiological nature of this work, no registration was necessary.

## Supporting information


**Table S1:** Search strategy.
**Table S2:** Classification of verbatims in conclusions.

## Data Availability

Data are available on request from the authors.

## References

[1] V. K. C. Yan , H. L. Li , L. Wei , M. R. J. Knapp , I. C. K. Wong , and E. W. Chan , “Evolving Trends in Consumption of Direct Oral Anticoagulants in 65 Countries/Regions From 2008 to 2019,” Drugs 83, no. 4 (2023): 315–340, 10.1007/s40265-023-01837-0. 36840892

[2] L. Staerk , E. L. Fosbøl , K. Gadsbøll , et al., “Non‐vitamin K Antagonist Oral Anticoagulation Usage According to Age Among Patients With Atrial Fibrillation: Temporal Trends 2011–2015 in Denmark,” Scientific Reports 6, no. 1 (2016): 31477, 10.1038/srep31477. PMC4980590 27510920

[3] S. Perreault , S. de Denus , B. White‐Guay , et al., “Oral Anticoagulant Prescription Trends, Profile Use, and Determinants of Adherence in Patients With Atrial Fibrillation,” Pharmacotherapy 40, no. 1 (2020): 40–54, 10.1002/phar.2350. 31758592

[4] J. Zhu , G. C. Alexander , S. Nazarian , J. B. Segal , and A. W. Wu , “Trends and Variation in Oral Anticoagulant Choice in Patients with Atrial Fibrillation, 2010‐2017,” Pharmacotherapy 38, no. 9 (2018): 907–920, 10.1002/phar.2158. PMC6448138 29920705

[5] S. Y. Loo , S. Dell'Aniello , L. Huiart , and C. Renoux , “Trends in the Prescription of Novel Oral Anticoagulants in UK Primary Care,” British Journal of Clinical Pharmacology 83, no. 9 (2017): 2096–2106, 10.1111/bcp.13299. PMC5555878 28390065

[6] Haute Autorité de Santé (HAS) , Rapport d'évaluation Des Médicaments Anticoagulants Oraux (Haute Autorité de Santé (HAS), 2018), https://www.has‐sante.fr/upload/docs/application/pdf/2018‐02/rapport_reev_aco_cteval234_2018‐02‐09_15‐38‐37_999.pdf.

[7] C. B. Granger , J. H. Alexander , J. J. V. McMurray , et al., “Apixaban Versus Warfarin in Patients With Atrial Fibrillation,” New England Journal of Medicine 365, no. 11 (2011): 981–992, 10.1056/NEJMoa1107039. 21870978

[8] R. P. Giugliano , C. T. Ruff , E. Braunwald , et al., “Edoxaban Versus Warfarin in Patients With Atrial Fibrillation,” New England Journal of Medicine 369, no. 22 (2013): 2093–2104, 10.1056/NEJMoa1310907. 24251359

[9] M. R. Patel , K. W. Mahaffey , J. Garg , et al., “Rivaroxaban Versus Warfarin in Nonvalvular Atrial Fibrillation,” New England Journal of Medicine 365, no. 10 (2011): 883–891, 10.1056/NEJMoa1009638. 21830957

[10] L. P. T. Joosten , S. van Doorn , P. M. van de Ven , et al., “Safety of Switching From a Vitamin K Antagonist to a Non‐Vitamin K Antagonist Oral Anticoagulant in Frail Older Patients With Atrial Fibrillation: Results of the FRAIL‐AF Randomized Controlled Trial,” Circulation 149, no. 4 (2024): 279–289, 10.1161/CIRCULATIONAHA.123.066485. 37634130

[11] T. van der Hulle , J. Kooiman , P. L. den Exter , O. M. Dekkers , F. A. Klok , and M. V. Huisman , “Effectiveness and Safety of Novel Oral Anticoagulants as Compared With Vitamin K Antagonists in the Treatment of Acute Symptomatic Venous Thromboembolism: A Systematic Review and Meta‐Analysis,” Journal of Thrombosis and Haemostasis 12, no. 3 (2014): 320–328, 10.1111/jth.12485. 24330006

[12] N. van Es , M. Coppens , S. Schulman , S. Middeldorp , and H. R. Büller , “Direct Oral Anticoagulants Compared With Vitamin K Antagonists for Acute Venous Thromboembolism: Evidence From Phase 3 Trials,” Blood 124, no. 12 (2014): 1968–1975, 10.1182/blood-2014-04-571232. 24963045

[13] S. V. Konstantinides , G. Meyer , C. Becattini , et al., “2019 ESC Guidelines for the Diagnosis and Management of Acute Pulmonary Embolism Developed in Collaboration With the European Respiratory Society (ERS): The Task Force for the Diagnosis and Management of Acute Pulmonary Embolism of the European Society of Cardiology (ESC),” European Heart Journal 41, no. 4 (2020): 543–603, 10.1093/eurheartj/ehz405. 31504429

[14] G. Hindricks , T. Potpara , N. Dagres , et al., “2020 ESC Guidelines for the Diagnosis and Management of Atrial Fibrillation Developed in Collaboration With the European Association for Cardio‐Thoracic Surgery (EACTS): The Task Force for the Diagnosis and Management of Atrial Fibrillation of the European Society of Cardiology (ESC) Developed With the Special Contribution of the European Heart Rhythm Association (EHRA) of the ESC,” European Heart Journal 42, no. 5 (2021): 373–498, 10.1093/eurheartj/ehaa612. 32860505

[15] I. C. Van Gelder , M. Rienstra , K. V. Bunting , et al., “2024 ESC Guidelines for the Management of Atrial Fibrillation Developed in Collaboration With the European Association for Cardio‐Thoracic Surgery (EACTS),” European Heart Journal 45, no. 36 (2024): 3314–3414, 10.1093/eurheartj/ehae176. 39210723

[16] J. Y. Jenny , A. Godier , C. Heim , S. Langenecker , E. Thienpont , and J. Eikelboom , “European Guidelines on Peri‐Operative Venous Thromboembolism Prophylaxis: First Update.: Chapter 13: Nonambulatory Orthopaedic Surgery,” European Journal of Anaesthesiology 41, no. 8 (2024): 622–626, 10.1097/EJA.0000000000002020. 38957031

[17] S. M. Stevens , S. C. Woller , L. B. Kreuziger , et al., “Antithrombotic Therapy for VTE Disease: Second Update of the CHEST Guideline and Expert Panel Report,” Chest 160, no. 6 (2021): e545–e608, 10.1016/j.chest.2021.07.055. 34352278

[18] “GRADE handbook,” accessed August 2, 2025, https://gdt.gradepro.org/app/handbook/handbook.html#h.3183vuv3ey12.

[19] “E 9 Statistical Principles for Clinical Trials,” (1998), https://www.ema.europa.eu/en/documents/scientific‐guideline/ich‐e‐9‐statistical‐principles‐clinical‐trials‐step‐5_en.pdf. 10.1177/030006059802600201 9602983

[20] M. Kicinski , “How Does Under‐Reporting of Negative and Inconclusive Results Affect the False‐Positive Rate in Meta‐Analysis? A Simulation Study,” BMJ Open 4, no. 8 (2014): e004831, 10.1136/bmjopen-2014-004831. PMC4156818 25168036

[21] R. Boussageon , C. Blanchard , E. Charuel , et al., “Project Rebuild the Evidence Base (REB): A Method to Interpret Randomised Clinical Trials and Their meta‐Analysis to Present Solid Benefit‐Risk Assessments to Patients,” Thérapie 78, no. 4 (2023): 353–365, 10.1016/j.therap.2022.10.008. 36371260

[22] “Covidence—Better Systematic Review Management,” Covidence. accessed September 28, 2025, https://www.covidence.org/.

[23] “AMSTAR ‐ Assessing the Methodological Quality of Systematic Reviews,” accessed August 2, 2025, https://amstar.ca/Amstar_Checklist.php.

[24] P. A. Games , “An Improved t Table for Simultaneous Control on g Contrasts,” Journal of the American Statistical Association 72, no. 359 (1977): 531–534, 10.1080/01621459.1977.10480607.

[25] H. Abdi , The Bonferonni and Šidák Corrections for Multiple Comparisons (Sage, 2007), https://personal.utdallas.edu/~herve/Abdi‐Bonferroni2007‐pretty.pdf.

[26] J. Thomas , L. M. Askie , J. A. Berlin , et al., “Chapter 22: Prospective Approaches to Accumulating Evidence (Last Updated October 2019),” in Cochrane Handbook for Systematic Reviews of Interventions Version 6.5, eds. J. P. Higgins , J. Thomas , J. Chandler , et al. (Cochrane, 2024. Available from), cochrane.org/handbook.

[27] J. A. López‐López , J. A. C. Sterne , H. H. Z. Thom , et al., “Oral Anticoagulants for Prevention of Stroke in Atrial Fibrillation: Systematic Review, Network Meta‐Analysis, and Cost Effectiveness Analysis,” BMJ 359 (2017): j5058, 10.1136/bmj.j5058. PMC5704695 29183961

[28] R. Bender and S. Lange , “Adjusting for Multiple Testing—When and How?,” Journal of Clinical Epidemiology 54, no. 4 (2001): 343–349, 10.1016/s0895-4356(00)00314-0. 11297884

[29] Q. Shi , E. S. Pavey , and R. E. Carter , “Bonferroni‐Based Correction Factor for Multiple, Correlated Endpoints,” Pharmaceutical Statistics 11, no. 4 (2012): 300–309, 10.1002/pst.1514. 22588983

[30] J. Ranstam , “Hypothesis‐Generating and Confirmatory Studies, Bonferroni Correction, and Pre‐Specification of Trial Endpoints,” Acta Orthopaedica 90, no. 4 (2019): 297, 10.1080/17453674.2019.1612624. PMC6718169 31084234

[31] M. Neuhäuser , “How to Deal With Multiple Endpoints in Clinical Trials,” Fundamental & Clinical Pharmacology 20, no. 6 (2006): 515–523, 10.1111/j.1472-8206.2006.00437.x. 17109645

[32] W. S. Browner and T. B. Newman , “Are All Significant P Values Created Equal? The Analogy Between Diagnostic Tests and Clinical Research,” Journal of the American Medical Association 257, no. 18 (1987): 2459–2463, 10.1001/jama.1987.03390180077027. 3573245

[33] F. Gueyffier and R. Boussageon , “Fondamentaux de l'évaluation des thérapeutiques, médicamenteuses et non médicamenteuses – un cadre pour l'évaluation des processus d'autoguérison,” Hegel 4, no. 4 (2023): 347–358, 10.3917/heg.134.0347.

[34] I. Boutron and P. Ravaud , “Misrepresentation and Distortion of Research in Biomedical Literature,” Proceedings of the National Academy of Sciences of the United States of America 115, no. 11 (2018): 2613–2619, 10.1073/pnas.1710755115. PMC5856510 29531025

[35] W. J. Sutherland , D. Spiegelhalter , and M. Burgman , “Policy: Twenty Tips for Interpreting Scientific Claims,” Nature 503, no. 7476 (2013): 335–337, 10.1038/503335a. 24273799

[36] I. Boutron , S. Dutton , P. Ravaud , and D. G. Altman , “Reporting and Interpretation of Randomized Controlled Trials With Statistically Nonsignificant Results for Primary Outcomes,” Journal of the American Medical Association 303, no. 20 (2010): 2058–2064, 10.1001/jama.2010.651. 20501928

[37] A. K. Reddy , S. Shepard , R. Ottwell , et al., “Over 30% of Systematic Reviews and Meta‐Analyses Focused on Rotator Cuff Tear Treatments Contained Spin in the Abstract,” Arthroscopy 37, no. 9 (2021): 2953–2959, 10.1016/j.arthro.2021.03.066. 33887409

[38] A. K. Reddy , K. Lulkovich , A. Wirtz , et al., “Assessment of Spin in the Abstracts of Systematic Reviews and Meta‐Analyses on Platelet‐Rich Plasma Treatment in Orthopaedics: A Cross‐Sectional Analysis,” Orthopaedic Journal of Sports Medicine 11, no. 2 (2023): 23259671221137923, 10.1177/23259671221137923. PMC9940191 36814771

[39] H. Flores , D. Kannan , R. Ottwell , et al., “Evaluation of Spin in the Abstracts of Systematic Reviews and meta‐Analyses on Breast cancer Treatment, Screening, and Quality of Life Outcomes: A Cross‐Sectional Study,” Journal of Cancer Policy 27 (2021): 100268, 10.1016/j.jcpo.2020.100268. 35559938

[40] T. R. Doyle , M. S. Davey , T. K. Moore , et al., “Most Systematic Reviews and Meta‐Analyses Reporting Clinical Outcomes of the Remplissage Procedure Have at Least 1 Form of Spin,” Arthrosc Sports Med Rehabil 6, no. 5 (2024): 100969, 10.1016/j.asmr.2024.100969. PMC11551395 39534027

[41] M. J. Page , J. E. McKenzie , P. M. Bossuyt , et al., “The PRISMA 2020 Statement: An Updated Guideline for Reporting Systematic Reviews,” BMJ 372 (2021): n71, 10.1136/bmj.n71. PMC8005924 33782057

[42] K. Chiu , Q. Grundy , and L. Bero , “‘Spin’ in Published Biomedical Literature: A Methodological Systematic Review,” PLOS Biology 15, no. 9 (2017): e2002173, 10.1371/journal.pbio.2002173. PMC5593172 28892482

[43] A. Yavchitz , P. Ravaud , D. G. Altman , et al., “A New Classification of Spin in Systematic Reviews and Meta‐Analyses Was Developed and Ranked According to the Severity,” Journal of Clinical Epidemiology 75 (2016): 56–65, 10.1016/j.jclinepi.2016.01.020. 26845744

[44] ChatGPT . “ChatGPT,” accessed October 6, 2025, https://chatgpt.com/fr‐FR.

[45] J. K. Kim , M. Chua , M. Rickard , and A. Lorenzo , “ChatGPT and Large Language Model (LLM) Chatbots: The Current State of Acceptability and a Proposal for Guidelines on Utilization in Academic Medicine,” Journal of Pediatric Urology 19, no. 5 (2023): 598–604, 10.1016/j.jpurol.2023.05.018. 37328321

[46] Biswas S. ChatGPT and the Future of Medical , “ChatGPT and the Future of Medical Writing,” Radiology 307, no. 2 (2023): e223312, 10.1148/radiol.223312. 36728748

