# Supplementary material for: Hypothetico‐Deductive Reasoning in Direct Oral Anticoagulant Trial Meta‐Analyses: A Meta‐Epidemiological Study
Source: Fundam Clin Pharmacol. 2026 Nov 13;40(6):e70118. doi: 10.1111/fcp.70118 (PMC13760596; doi:10.1111/fcp.70118)
Supplement: Supplementary file 1 [file FCP-40-0-s001.docx]

**Table S1.** Search strategy

PUBMED:

(("anticoagulants"[MeSH Terms] OR "apixaban"[Title/Abstract] OR "rivaroxaban"[Title/Abstract] OR "edoxaban"[Title/Abstract] OR "dabigatran"[Title/Abstract]) AND ("atrial fibrillation*"[MeSH Terms] OR "thromboembolism*"[MeSH Terms] OR "pulmonary embolism"[MeSH Terms] OR ("deep"[All Fields] AND "venous thrombos*"[MeSH Terms]) OR (("deep"[All Fields] AND ("veins"[MeSH Terms] OR "veins"[All Fields] OR "vein"[All Fields])) AND "thrombos*"[MeSH Terms]) OR "arthroplasty, replacement, knee"[MeSH Terms] OR "arthroplasty, replacement, hip"[MeSH Terms] OR "arthroplasty, replacement, hip"[MeSH Terms])) AND (meta-analysis[Filter]) NOT ("network meta analysis"[MeSH Terms]) AND (clinical trials, randomized[MeSH Terms])

Embase:

('atrial fibrillation'/exp OR 'thromboembolism'/exp OR 'lung embolism'/exp OR 'total hip prosthesis'/exp OR 'total hip replacement'/exp OR 'total knee arthroplasty'/exp OR 'total knee prosthesis'/exp) AND ('apixaban'/exp OR 'rivaroxaban'/exp OR 'dabigatran'/exp OR 'edoxaban'/exp) AND ('placebo'/exp OR 'warfarin'/exp OR 'anticoagulant agent'/exp) AND ('prophylaxis'/exp OR 'drug therapy'/exp OR 'side effect'/exp) AND 'meta analysis'/exp AND 'randomized controlled trial'/exp

**Table S2.** Classification of verbatims in conclusions

| **Confirmatory statements** | **Exploratory statements** |
| --- | --- |
| *confirmed* | *suggested* |
| *proved* | *suggests* |
| *provided evidence for* | *was in favor of* |
| *resulted in* | *was consistent with* |
| *led to* | *appeared to* |
| *was associated with* | *may* |
| *indicated* | *might* |
| *strongly supported* | *was compatible with* |
| *indicate that DOAC are more or similarly* | *pointed to* |
| *can therefore feel more confident* | *implied* |
|  | *raised the possibility that* |
|  | *was aligned with* |
|  | *within the limitations* |
|  | *tend to corroborate* |
|  | *should be considered* |
|  | *more evidence is needed to verify* |

# Appendix A

## Protocol

We here reported the protocol following PRISMA-P (Preferred Reporting Items for Systematic review and Meta-Analysis Protocols) 2015 checklist <https://www.equator-network.org/reporting-guidelines/prisma-protocols/> .

**Appendix 1.** Protocol (continued on next pages)

| **Section and topic** | **Item No** | **Checklist item completion** |
| --- | --- | --- |
| Title: |  |  |
| Identification | 1a | Estimating the Risk of False Positives in Meta-Analyses: A Review of Hypothetico-Deductive Reasoning in Systematic Reviews of Direct Oral Anticoagulants |
| Update | 1b | The protocol is not an update |
| Registration | 2 | The protocol has been registered on OSF: [osf.io/8tjx9](http://osf.io/8tjx9) |
| Authors: |  |  |
| Contact | 3a | Q Remy, A Gougeon^1,^ 2, P Riallin, Remy Boussageon,^1^ JC Lega^1, 3, 4^  ^1^ Laboratoire de Biométrie et Biologie Evolutive UMR 5558, CNRS, Université Lyon 1, Université de Lyon, Villeurbanne, France  ^2^ RCTs, Lyon, France  ^3^ Service de Rhumatologie, Hôpital Lyon Sud, Hospices Civils de Lyon, Lyon, France  ^4^ Service Hospitalo-Universitaire de Pharmacotoxicologie, Pôle Santé Publique, Hospices Civils de Lyon, Lyon, France  Correspondence to: Université Lyon 1  E-mail: jean-christophe.lega@chu-lyon.fr |
| Contributions | 3b | QR drafted the protocol, PR is the second reviewer, RB, JCL, AG revised the protocol, AG and JCL supervised and designed the methodology and are the guarantors of the review. |
| Amendments | 4 | This protocol is based on a previous template but is not a formal amendment of an existing registered protocol |
| Support: |  |  |
| Sources | 5a | The Biometry and Evolutionary Biology laboratory (LBBE), Unit 5558 University of Claude Bernard Lyon 1 |
| Sponsor | 5b | NA |
| Role of sponsor or funder | 5c | NA |
| Rationale | 6 | Since their introduction in 2008, direct oral anticoagulants (DOACs) have become a cornerstone in the prevention and treatment of thromboembolic diseases, particularly in non-valvular atrial fibrillation (NVAF), venous thromboembolism (VTE), and postoperative thromboprophylaxis following major orthopedic surgery. Current guidelines from the European Society of Cardiology (ESC) and the French Society of Anesthesia and Resuscitation (SFAR) strongly support the use of DOACs in these indications. However, the robustness of the underlying these recommendations relies significantly on meta-analyses, which, despite their methodological advantages, are prone to several biases.  Meta-analyses synthesizing randomized controlled trials (RCTs) are frequently used to inform clinical guidelines, yet they are not immune to methodological pitfalls that can lead to false positives—incorrectly concluding a treatment effect when none exists. Such errors may arise due to several factors   - **Risk of false positives:** Meta-analyses may produce false positives due to biases such as selective reporting, publication bias, and the inclusion of randomized controlled trials (RCTs) at high risk of bias, as the false positive risk propagates to the meta-analysis— following the concept of garbage in, garbage out (GIGO). These erroneous findings can misinform clinical practice and guideline development. - **Hypothetico-deductive reasoning:** This approach involves forming hypotheses and testing them through observation and experimentation. In the context of antithrombotic therapy, systematically analyzing whether the endpoints are 'exploratory' (hypothesis-generating) or 'confirmatory' (hypothesis-testing), with pre-specified criteria and consideration of multiplicity, can aid in evaluating the evidence and determining the robustness of clinical trial findings. The assessment of the hypothetico-deductive approach is lacking in the GRADE tool for determining evidence levels. The Rebuild the Evidence Based (REB) tool developed by Pr Boussageon and colleagues is designed to assess the reality of the hypothetico-deductive approach. |
| Objectives | 7 | The aim of this review is to evaluate the risk of false positives in direct oral anticoagulants meta-analyses by evaluating the contribution of study design and statistical methods to these errors, and to examine how the application of hypothetico-deductive reasoning can improve the reliability of conclusions in these analyses. |
| Eligibility criteria | 8 | PICOT: P: Patients: humans over 18 years old, with NVAF or PE or VTE or post total hip, knee replacement; I: Intervention: DOACs (apixaban, rivaroxaban, edoxaban, dabigatran) (any); C: Control active (including DOACs, vitamin K antagonist, low-molecular-weight-heparin (LMWH)), aspirin or placebo; O: Outcome: efficacy (thrombosis) OR safety (bleeding) meta-analytical outcome T: Type of study: meta-analysis of phase III trials  Main exclusion criteria will be:  - Pediatric populations (under 18 years old)  - Non-anticoagulant treatments or non-pharmacological strategies, not approved DOACs (EMA or FDA)  - Meta-analyses comparing a control to grouped anticoagulant therapies broadly defined to encompass all classes, or conducted almost exclusively on subgroup analyses and not assessing the overall comparison between DOACs and control.  - off-label uses of DOAC  - Observational data, network meta-analyses |
| Information sources | 9 | Articles published in Pubmed or Embase since 1994, published in English; no grey literature sources will be used. |
| Search strategy | 10 | Combination of a different keywords for each parameter (antithrombotic therapy, outcome) (appendix 1) |
| Study records: |  |  |
| Data management | 11a | The extracted data will be managed in a structured format to ensure transparency and reproducibility.   - **Selection of meta-analyses**: The included studies will be screened and managed using the online Covidence tool, where the selection process will be documented. - **Data organization**: Information relevant to the analysis of false-positive risks in meta-analyses will be extracted and stored systematically. - **Storage and access**: The data will be archived in a secure, cloud-based repository or an institutional storage system for long-term accessibility. - **Synthesis and analysis**: The extracted data will be used to assess how statistical approaches influence the estimation of false-positive risks.. |
| Selection process | 11b | Titles and abstracts of studies retrieved using the search strategy will be screened independently by two review authors to identify studies that potentially meet the inclusion criteria outlined above. The full text of these potentially eligible studies will be retrieved and independently assessed for eligibility by the two same review authors. Any disagreement between them over the eligibility of particular studies will be resolved through discussion with a third reviewer.  In case the number of eligible meta-analyses exceeds 120, a random sample of studies will be drawn for data extraction. This approach is intended to maintain feasibility while preserving representativeness.  Data will then be extracted from the studies selected for inclusion as follows. |
| Data collection process | 11c | Two reviewers for data extraction (QR, PR). Data will be collected manually. |
| Data items | 12 | The following variables will be extracted from the selected meta-analyses:  Descriptive data:  - Title of the study, PMID, DOI  - Authors and publication year  - Journal  - Type of anticoagulant oral direct (AOD) studied  - Type of comparator (e.g., warfarin, placebo)  - Disease studied (e.g., atrial fibrillation, venous thromboembolism)  - Number of included primary studies per meta-analysis  - Number of included participants per meta-analysis  Quantitative data:  - Number of statistical tests performed: only tests aiming to compare groups based on a clinical outcome (efficacy or safety), or to assess the significance of interactions, were counted. Tests of heterogeneity (I², Cochran’s Q) or descriptive analyses were not considered as potential sources of false positives, as they do not lead to direct clinical conclusions.  - Significance level (α) used in the analyses  Qualitative data:  - Presence of an explicit hypothesis (expected differences between molecules on the primary endpoint)  - Presence of at least one positive trial supporting the hypothesis  - Presence of a pre-specified protocol  - Exploratory vs. confirmatory nature of the reported results: basically using a verbatim approach, we will count the number of confirmatory or exploratory statements related to the main hypothesis (or, if not explicitly stated, the research question) in the conclusion section of the meta-analysis.  - Management of alpha risk and multiplicity of tests (alpha risk adjustment reported, hierarchical testing strategy…)  - Presence of a sensitivity analysis and a risk of bias assessment (leave-one-out, exclusion of phase II trials, class effect analysis) |
| Outcomes and prioritization | 13 | **False Positive Risk Calculation related to number of statistical tests**: We will calculate the risk of false positives using the following formula:  P (at least one false positive)=1−(1−α)^n^  Where:  α: The significance level for type 1 error for each test (typically 0.05).  n: The number of independent statistical tests performed for each meta-analysis.  For instance, if a meta-analysis conducts 20 independent tests with α=0.05, the cumulative risk of at least one false positive is: P(at least one false positive)=1−(0.95)^20^≈0.642  **Qualitative Analysis**: We will analyze the discussions and conclusion in the meta-analyses to identify how authors address hypothesis formation, testing, the integration of confirmatory trials (i.e. trial with positive results for primary outcome) and concluding (evidence or not). |
| Risk of bias in individual studies | 14 | The risk of bias in the included meta-analyses will be evaluated based on methodological and statistical criteria relevant to the estimation of false-positive risk. Specifically, we will assess:   - **Statistical approach**: Whether the meta-analysis applied corrections for multiple testing and alpha risk control. - **Exploratory vs. confirmatory nature**: Whether the statistical plan was predefined or results were primarily hypothesis-generating. - **Reproducibility and transparency**: Whether the meta-analysis provided sufficient details on statistical methods, study selection criteria, and sensitivity analyses. - **Publication bias assessment**: Whether the authors conducted a publication bias analysis (e.g., funnel plot, Egger’s test).   No formal risk of bias tool will be applied, as the objective of this review is not to evaluate the clinical validity of individual trials, but to analyze how statistical risk is discussed and managed within meta-analyses. However, we will extract whether authors reported using a risk of bias assessment tool (e.g., RoB 2, NOS), and whether this assessment was used to conduct sensitivity analyses (e.g., exclusion of high-risk studies). The way such assessments are integrated into the interpretation of findings will also be described. |
| Data synthesis | 15a | Quantitative synthesis will be conducted only for meta-analyses that report the number of statistical tests performed and the significance level (α) used. These criteria are required to compute the cumulative risk of false positives. Studies not reporting these two variables will be excluded from the analysis**.** |
|  | 15b | The main summary measure will be the estimated cumulative risk of false positives, calculated using the formula:P (at least one false positive)=1−(1−α)^n^  where **α** is the significance threshold and *n* is the number of statistical tests reported in the meta-analysis.  No meta-analytic pooling of effect sizes will be performed, as the objective is not to produce a combined clinical estimate but to assess statistical robustness.  Heterogeneity between studies will not be quantitatively assessed using I² or Kendall’s τ, since the unit of analysis is not clinical effect size but methodological characteristics. |
|  | 15c | No statistical subgroup analyses, sensitivity analyses, or meta-regression are planned. However, descriptive comparisons will be made across meta-analyses regarding key methodological features such as the use of a statistical analysis plan, the presence of a predefined hypothesis, and the handling of alpha risk and multiplicity. |
|  | 15d | As this review does not aim to pool clinical outcomes or effect sizes, all included meta-analyses will undergo a structured qualitative synthesis. The analysis will focus on how statistical results are presented, whether conclusions are framed in confirmatory or exploratory terms, and whether methodological safeguards (e.g., predefined hypotheses, alpha risk control) are reported. When sufficient data are available, a complementary estimation of the cumulative false-positive risk will be calculated. |
| Meta-bias(es) | 16 | Meta-bias assessment is not explicitly conducted as our focus is on the statistical properties of included meta-analyses rather than biases at the primary study level |
| Confidence in cumulative evidence | 17 | Confidence in cumulative evidence assessment is not performed as we are not synthesizing clinical outcomes but rather methodological trends across meta-analyses |

## APPENDIX B

## References of included studies

1.	Abatzis-Papadopoulos M, Tigkiropoulos K, Nikas S, Papoutsis I, Kostopoulou O, Stavridis K, et al. The effectiveness and safety of direct oral anticoagulants compared to conventional pharmacologic thromboprophylaxis in hip fracture patients: A systematic review and meta-analysis of randomized controlled trials. Orthopaedics & Traumatology: Surgery & Research. avr 2023;109(2):103364.

2.	Ahmed M, Ahsan A, Shafiq A, Ahmed R, Alam M, Sabouret P, et al. Meta‐Analysis Comparing Oral Anticoagulant Monotherapy Versus Dual Antithrombotic Therapy in Patients With Atrial Fibrillation and Stable Coronary Artery Disease. Clinical Cardiology. Wiley; oct 2024;47(10).

3.	Andò G, Trio O, Carerj S. New oral anticoagulants versus vitamin K antagonists before cardioversion of atrial fibrillation: a meta-analysis of data from 4 randomized trials. Expert Review of Cardiovascular Therapy. 4 mai 2015;13(5):577‑83.

4.	As-Sultany M, Pagkalos J, Yeganeh S, Craigs CL, Korres N, West RM, et al. Use of Oral Direct Factor Xa Inhibiting Anticoagulants in Elective Hip and Knee Arthroplasty: A Meta-analysis of Efficacy and Safety Profiles Compared with those of Low-Molecular-Weight Heparins. CVP. 1 mai 2013;11(3):366‑75.

5.	Bennaghmouch N, De Veer AJWM, Bode K, Mahmoodi BK, Dewilde WJM, Lip GYH, et al. Efficacy and Safety of the Use of Non–Vitamin K Antagonist Oral Anticoagulants in Patients With Nonvalvular Atrial Fibrillation and Concomitant Aspirin Therapy: A Meta-Analysis of Randomized Trials. Circulation. 13 mars 2018;137(11):1117‑29.

6.	Bloom BJ, Filion KB, Atallah R, Eisenberg MJ. Meta-Analysis of Randomized Controlled Trials on the Risk of Bleeding With Dabigatran. The American Journal of Cardiology. mars 2014;113(6):1066‑74.

7.	Boonyawat K, Caron F, Li A, Chai‐Adisaksopha C, Lim W, Iorio A, et al. Association of body weight with efficacy and safety outcomes in phase III randomized controlled trials of direct oral anticoagulants: a systematic review and meta‐analysis. Journal of Thrombosis and Haemostasis. juill 2017;15(7):1322‑33.

8.	Brunetti ND, Tricarico L, Correale M, De Gennaro L, Santoro F, Ieva R, et al. Direct oral anticoagulants more effective than low-molecular-weight heparin for venous thrombo-embolism in cancer: an updated meta-analysis of randomized trials. J Thromb Thrombolysis. août 2020;50(2):305‑10.

9.	Brunetti ND, Tricarico L, Tilz RR, Heeger CH, De Gennaro L, Correale M, et al. Lower Major Bleeding Rates with Direct Oral Anticoagulants in Catheter Ablation of Atrial Fibrillation: an Updated Meta-analysis of Randomized Controlled Studies. Cardiovasc Drugs Ther. avr 2020;34(2):209‑14.

10.	Caldeira D, Barra M, Ferreira A, Rocha A, Augusto A, Pinto FJ, et al. Systematic review with meta‐analysis: the risk of major gastrointestinal bleeding with non‐vitamin K antagonist oral anticoagulants. Aliment Pharmacol Ther. déc 2015;42(11‑12):1239‑49.

11.	Caldeira D, Canastro M, Barra M, Ferreira A, Costa J, Pinto FJ, et al. Risk of Substantial Intraocular Bleeding With Novel Oral Anticoagulants: Systematic Review and Meta-analysis. JAMA Ophthalmol. 1 juill 2015;133(7):834.

12.	Caldeira D, Gonçalves N, Ferreira JJ, Pinto FJ, Costa J. Tolerability and Acceptability of Non-Vitamin K Antagonist Oral Anticoagulants in Atrial Fibrillation: Systematic Review and Meta-Analysis. Am J Cardiovasc Drugs. Springer Science and Business Media LLC; août 2015;15(4):259‑65.

13.	Cao YB, Zhang JD, Shen H, Jiang YY. Rivaroxaban versus enoxaparin for thromboprophylaxis after total hip or knee arthroplasty: a meta-analysis of randomized controlled trials. Eur J Clin Pharmacol. nov 2010;66(11):1099‑108.

14.	Capodanno D, Capranzano P, Giacchi G, Calvi V, Tamburino C. Novel oral anticoagulants versus warfarin in non-valvular atrial fibrillation: A meta-analysis of 50,578 patients. International Journal of Cardiology. août 2013;167(4):1237‑41.

15.	Carmo J, Ferreira J, Costa F, Carmo P, Cavaco D, Carvalho S, et al. Non-vitamin K antagonist oral anticoagulants compared with warfarin at different levels of INR control in atrial fibrillation: A meta-analysis of randomized trials. International Journal of Cardiology. oct 2017;244:196‑201.

16.	Casula M, Fortuni F, Fabris F, Leonardi S, Gnecchi M, Sanzo A, et al. Direct oral Xa inhibitors versus warfarin in patients with cancer and atrial fibrillation: a meta-analysis. Journal of Cardiovascular Medicine. août 2020;21(8):570‑6.

17.	Chai‐Adisaksopha C, Hillis C, Isayama T, Lim W, Iorio A, Crowther M. Mortality outcomes in patients receiving direct oral anticoagulants: a systematic review and meta‐analysis of randomized controlled trials. Journal of Thrombosis and Haemostasis. nov 2015;13(11):2012‑20.

18.	Chaudhary R, Pagali S, Garg J, Murad MH, Wysokinski WE, McBane RD. DOACs Versus VKAs in Older Adults Treated for Acute Venous Thromboembolism: Systematic Review and Meta‐Analysis. J American Geriatrics Society. sept 2020;68(9):2021‑6.

19.	Chen HB, Xiu J, Li YH, Yu TH. The risk of bleeding and all-cause mortality with edoxaban versus vitamin K antagonists: A meta-analysis of phase III randomized controlled trials. Thrombosis Research. oct 2020;194:82‑90.

20.	Chen X, Guo L, Lin M. Efficacy and Safety of Direct Oral Anticoagulants in Cerebral Venous Thrombosis: Meta-Analysis of Randomized Clinical Trials. Clin Appl Thromb Hemost. janv 2024;30:10760296241256360.

21.	Costello M, Murphy R, Judge C, Ruttledge S, Gorey S, Loughlin E, et al. Effect of non‐vitamin‐K oral anticoagulants on stroke severity compared to warfarin: a meta‐analysis of randomized controlled trials. Euro J of Neurology. mars 2020;27(3):413‑8.

22.	Coyle M, Lynch A, Higgins M, Costello M, Judge C, O’Donnell M, et al. Risk of Intracranial Hemorrhage Associated With Direct Oral Anticoagulation vs Antiplatelet Therapy: A Systematic Review and Meta-Analysis. JAMA Netw Open. 4 déc 2024;7(12):e2449017.

23.	De Lucena LA, Freitas MAA, Souza AKC, Silva CHA, Watanabe JMF, Guedes FL, et al. Direct oral anticoagulants versus vitamin K antagonists in patients with atrial fibrillation and stage 5 chronic kidney disease under dialysis: A systematic review and meta-analysis of randomized controlled trials. J Thromb Thrombolysis. 28 janv 2024;57(3):381‑9.

24.	Dentali F, Botto GL, Gianni M, Ambrosino P, Di Minno MND. Efficacy and safety of direct oral anticoagulants in patients undergoing cardioversion for atrial fibrillation: A systematic review and meta-analysis of the literature. International Journal of Cardiology. avr 2015;185:72‑7.

25.	Dentali F, Di Minno MND, Gianni M, Ambrosino P, Squizzato A, Ageno W. Non-vitamin K oral anticoagulants in patients with pulmonary embolism: a systematic review and meta-analysis of the literature. Intern Emerg Med. juin 2015;10(4):507‑14.

26.	Dentali F, Sironi A, Gianni M, Orlandini F, Guasti L, Grandi A, et al. Gender Difference in Efficacy and Safety of Nonvitamin K Antagonist Oral Anticoagulants in Patients with Nonvalvular Atrial Fibrillation or Venous Thromboembolism: A Systematic Review and a Meta-Analysis of the Literature. Semin Thromb Hemost. 26 sept 2015;41(07):774‑87.

27.	Desai A, Escamilla-Ocanas C, Dilip D, Saber H, Damani R. Risk of Stroke vs. Intracerebral Hemorrhage in Patients with Non-Valvular Atrial Fibrillation Undergoing Percutaneous Coronary Intervention: A Systematic Review and Meta-Analysis of Randomized Controlled Trials Comparing Dual vs. Triple Antithrombotic Therapy. Journal of Stroke and Cerebrovascular Diseases. Elsevier BV; avr 2021;30(4):105654.

28.	Di MInno MND, Ambrosino P. Direct oral anticoagulants for the treatment of unprovoked venous thromboembolism: a meta-analysis of randomised controlled trials. Blood Transfusion. 2014;

29.	Di Minno MND, Lupoli R, Di Minno A, Ambrosino P, Scalera A, Dentali F. Effect of body weight on efficacy and safety of direct oral anticoagulants in the treatment of patients with acute venous thromboembolism: A meta-analysis of randomized controlled trials. Annals of Medicine. 2 janv 2015;47(1):61‑8.

30.	Di Monaco A, Guida P, Vitulano N, Quadrini F, Troisi F, Langialonga T, et al. Catheter ablation of atrial fibrillation with uninterrupted anticoagulation: a meta-analysis of six randomized controlled trials. Journal of Cardiovascular Medicine. Ovid Technologies (Wolters Kluwer Health); juill 2020;21(7):483‑90.

31.	Doni K, Bühn S, Weise A, Mann NK, Hess S, Sönnichsen A, et al. Safety outcomes of direct oral anticoagulants in older adults with atrial fibrillation: a systematic review and meta-analysis of (subgroup analyses from) randomized controlled trials. GeroScience. Springer Science and Business Media LLC; 1 juin 2023;46(1):923‑44.

32.	Elbadawi A, Shnoda M, Mahmoud K, Elgendy IY. Efficacy and safety of direct oral anticoagulants vs. low molecular weight heparin for cancer-related venous thromboembolism: a meta-analysis of randomized trials. European Heart Journal - Cardiovascular Pharmacotherapy. Oxford University Press (OUP); 21 sept 2021;7(5):380‑8.

33.	Erdem G, Esme M, Doğu BB. Systematic review and meta-analysis of randomized controlled trials on safety and effectiveness of oral anticoagulants for atrial fibrillation in older people. Ir J Med Sci. Springer Science and Business Media LLC; déc 2022;191(6):2517‑23.

34.	Giustozzi M, Agnelli G, Del Toro-Cervera J, Klok FA, Rosovsky RP, Martin AC, et al. Direct Oral Anticoagulants for the Treatment of Acute Venous Thromboembolism Associated with Cancer: A Systematic Review and Meta-Analysis. Thromb Haemost. Georg Thieme Verlag KG; juill 2020;120(07):1128‑36.

35.	Gómez-Outes A, Lecumberri R, Suárez-Gea ML, Terleira-Fernández AI, Monreal M, Vargas-Castrillón E. Case Fatality Rates of Recurrent Thromboembolism and Bleeding in Patients Receiving Direct Oral Anticoagulants for the Initial and Extended Treatment of Venous Thromboembolism: A Systematic Review. J Cardiovasc Pharmacol Ther. sept 2015;20(5):490‑500.

36.	Huang HF, Li SS, Yang XT, Xie Q, Tian XB. Rivaroxaban versus enoxaparin for the prevention of venous thromboembolism after total knee arthroplasty: A meta-analysis. Medicine. Ovid Technologies (Wolters Kluwer Health); nov 2018;97(48):e13465.

37.	Huang J, Liao C, Wu L, Cao Y, Gao F. Apixaban versus enoxaparin in patients with total knee arthroplasty: A meta-analysis of randomised trials. Thromb Haemost. Georg Thieme Verlag KG; 2011;105(02):245‑53.

38.	Kahale LA, Hakoum MB, Tsolakian IG, Alturki F, Matar CF, Terrenato I, et al. Anticoagulation for the long-term treatment of venous thromboembolism in people with cancer. Cochrane Gynaecological, Neuro-oncology and Orphan Cancer Group, rédacteur. Cochrane Database of Systematic Reviews. 19 juin 2018;2023(1).

39.	Karatisidis L, Zagoridis K, Mprotsis T, Misidou C, Pentidou A, Vrachiolias G, et al. Obese patients with atrial fibrillation are more efficiently protected from thrombosis under warfarin or xabans compared to non-obese patients; a systematic review and Meta-analysis of six randomized controlled trials. Thrombosis Research. Elsevier BV; juin 2024;238:88‑96.

40.	Kim IS, Kim HJ, Kim TH, Uhm JS, Joung B, Lee MH, et al. Appropriate doses of non-vitamin K antagonist oral anticoagulants in high-risk subgroups with atrial fibrillation: Systematic review and meta-analysis. Journal of Cardiology. Elsevier BV; oct 2018;72(4):284‑91.

41.	Kimachi M, Furukawa TA, Kimachi K, Goto Y, Fukuma S, Fukuhara S. Direct oral anticoagulants versus warfarin for preventing stroke and systemic embolic events among atrial fibrillation patients with chronic kidney disease. Cochrane Kidney and Transplant Group, rédacteur. Cochrane Database of Systematic Reviews. 6 nov 2017;2017(11).

42.	Kirkilesis GI, Kakkos SK, Tsolakis IA. Editor’s Choice – A Systematic Review and Meta-Analysis of the Efficacy and Safety of Anticoagulation in the Treatment of Venous Thromboembolism in Patients with Cancer. European Journal of Vascular and Endovascular Surgery. Elsevier BV; mai 2019;57(5):685‑701.

43.	Kumar S, Danik SB, Altman RK, Barrett CD, Lip GYH, Chatterjee S, et al. Non-Vitamin K Antagonist Oral Anticoagulants and Antiplatelet Therapy for Stroke Prevention in Patients With Atrial Fibrillation: A Meta-Analysis of Randomized Controlled Trials. Cardiology in Review. Ovid Technologies (Wolters Kluwer Health); sept 2016;24(5):218‑23.

44.	Larsen TB, Nielsen PB, Skjøth F, Rasmussen LH, Lip GYH. Non-Vitamin K Antagonist Oral Anticoagulants and the Treatment of Venous Thromboembolism in Cancer Patients: A Semi Systematic Review and Meta-Analysis of Safety and Efficacy Outcomes. Fukumoto Y, rédacteur. PLoS ONE. Public Library of Science (PLoS); 5 déc 2014;9(12):e114445.

45.	Lega JC, Bertoletti L, Gremillet C, Chapelle C, Mismetti P, Cucherat M, et al. Consistency of Safety and Efficacy of New Oral Anticoagulants across Subgroups of Patients with Atrial Fibrillation. PLOS ONE. Public Library of Science; 12 mars 2014;9(3):e91398.

46.	Liew A, O’Donnell M, Douketis J. Comparing mortality in patients with atrial fibrillation who are receiving a direct‐acting oral anticoagulant or warfarin: a meta‐analysis of randomized trials. Journal of Thrombosis and Haemostasis. sept 2014;12(9):1419‑24.

47.	Loffredo L, Perri L, Violi F. Impact of new oral anticoagulants on gastrointestinal bleeding in atrial fibrillation: A meta-analysis of interventional trials. Digestive and Liver Disease. mai 2015;47(5):429‑31.

48.	Ma G, Zhang R, Wu X, Wang D, Ying K. Direct factor Xa inhibitors (rivaroxaban and apixaban) versus enoxaparin for the prevention of venous thromboembolism after total knee replacement: A meta-analysis of 6 randomized clinical trials. Thrombosis Research. 1 mai 2015;135(5):816‑22.

49.	Makam RCP, Hoaglin DC, McManus DD, Wang V, Gore JM, Spencer FA, et al. Efficacy and safety of direct oral anticoagulants approved for cardiovascular indications: Systematic review and meta-analysis. PLOS ONE. Public Library of Science; 24 mai 2018;13(5):e0197583.

50.	Mareev VYu, Mareev YuV. Role of anticoagulants in therapy and prevention of recurrent venous thromboembolism in patients with cancer: a meta-analysis of randomized trials with apixaban. Kardiologiia. 31 mars 2022;62(3):4‑15.

51.	Marik PE, Cavallazzi R. Extended Anticoagulant and Aspirin Treatment for the Secondary Prevention of Thromboembolic Disease: A Systematic Review and Meta-Analysis. Gándara E, rédacteur. PLoS ONE. 20 nov 2015;10(11):e0143252.

52.	McIntyre WF, Benz AP, Becher N, Healey JS, Granger CB, Rivard L, et al. Direct Oral Anticoagulants for Stroke Prevention in Patients With Device-Detected Atrial Fibrillation: A Study-Level Meta-Analysis of the NOAH-AFNET 6 and ARTESiA Trials. Circulation. Ovid Technologies (Wolters Kluwer Health); 26 mars 2024;149(13):981‑8.

53.	Miller CS, Grandi SM, Shimony A, Filion KB, Eisenberg MJ. Meta-Analysis of Efficacy and Safety of New Oral Anticoagulants (Dabigatran, Rivaroxaban, Apixaban) Versus Warfarin in Patients With Atrial Fibrillation. The American Journal of Cardiology. Elsevier BV; août 2012;110(3):453‑60.

54.	Murphy AC, Koshy AN, Farouque O, Yeo B, Raman J, Kearney L, et al. Factor Xa Inhibition for the Treatment of Venous Thromboembolism Associated With Cancer: A Meta-Analysis of the Randomised Controlled Trials. Heart, Lung and Circulation. Elsevier BV; mai 2022;31(5):716‑25.

55.	Navalha DDP, Felix N, Nogueira A, Clemente M, Marinho AD, Ferreira ROM, et al. Direct oral anticoagulants versus vitamin K antagonists in patients with atrial fibrillation on chronic hemodialysis: a meta-analysis of randomized controlled trials. Int Urol Nephrol. Springer Science and Business Media LLC; 28 déc 2023;56(6):2001‑10.

56.	Nazha B, Pandya B, Cohen J, Zhang M, Lopes RD, Garcia DA, et al. Periprocedural Outcomes of Direct Oral Anticoagulants Versus Warfarin in Nonvalvular Atrial Fibrillation: Meta-Analysis of Phase III Trials. Circulation. Ovid Technologies (Wolters Kluwer Health); 2 oct 2018;138(14):1402‑11.

57.	Ning GZ, Kan SL, Chen LX, Shangguan L, Feng SQ, Zhou Y. Rivaroxaban for thromboprophylaxis after total hip or knee arthroplasty: a meta-analysis with trial sequential analysis of randomized controlled trials. Sci Rep. 29 mars 2016;6:23726.

58.	Ntaios G, Papavasileiou V, Diener HC, Makaritsis K, Michel P. Nonvitamin-K-antagonist oral anticoagulants versus warfarin in patients with atrial fibrillation and previous stroke or transient ischemic attack: An updated systematic review and meta-analysis of randomized controlled trials. International Journal of Stroke. SAGE Publications; août 2017;12(6):589‑96.

59.	Ntaios G, Papavasileiou V, Diener HC, Makaritsis K, Michel P. Nonvitamin-K-Antagonist Oral Anticoagulants in Patients With Atrial Fibrillation and Previous Stroke or Transient Ischemic Attack: A Systematic Review and Meta-Analysis of Randomized Controlled Trials. Stroke. déc 2012;43(12):3298‑304.

60.	Pathak R, Giri S, Karmacharya P, Aryal MR, Poudel DR, Ghimire S, et al. Meta-analysis on efficacy and safety of new oral anticoagulants for venous thromboembolism prophylaxis in elderly elective postarthroplasty patients. Blood Coagulation & Fibrinolysis. Ovid Technologies (Wolters Kluwer Health); déc 2015;26(8):934‑9.

61.	Patti G, Di Gioia G, Cavallari I, Nenna A. Safety and efficacy of nonvitamin K antagonist oral anticoagulants versus warfarin in diabetic patients with atrial fibrillation: A study‐level meta‐analysis of phase III randomized trials. Diabetes Metabolism Res. mars 2017;33(3):e2876.

62.	Plitt A, Zelniker TA, Park JG, McGuire DK, Ruff CT, Antman EM, et al. Patients with diabetes mellitus and atrial fibrillation treated with non-vitamin K antagonist oral anticoagulants: meta-analysis of eight outcomes in 58 634 patients across four randomized controlled trials. European Heart Journal - Cardiovascular Pharmacotherapy. Oxford University Press (OUP); 9 avr 2021;7(FI1):f40‑9.

63.	Robertson L, Kesteven P, McCaslin JE. Oral direct thrombin inhibitors or oral factor Xa inhibitors for the treatment of pulmonary embolism. Cochrane Vascular Group, rédacteur. Cochrane Database of Systematic Reviews. 4 déc 2015;2016(12).

64.	Robertson L, Yeoh SE, Ramli A. Secondary prevention of recurrent venous thromboembolism after initial oral anticoagulation therapy in patients with unprovoked venous thromboembolism. Cochrane Vascular Group, rédacteur. Cochrane Database of Systematic Reviews. 15 déc 2017;2017(12).

65.	Romero J, Cerrud-Rodriguez RC, Alviz I, Diaz JC, Rodriguez D, Arshad S, et al. Significant Benefit of Uninterrupted DOACs Versus VKA During Catheter Ablation of Atrial Fibrillation. JACC: Clinical Electrophysiology. Elsevier BV; déc 2019;5(12):1396‑405.

66.	Ruff CT, Giugliano RP, Braunwald E, Hoffman EB, Deenadayalu N, Ezekowitz MD, et al. Comparison of the efficacy and safety of new oral anticoagulants with warfarin in patients with atrial fibrillation: a meta-analysis of randomised trials. The Lancet. Elsevier BV; mars 2014;383(9921):955‑62.

67.	Sardar P, Chatterjee S, Chaudhari S, Lip GYH. New Oral Anticoagulants in Elderly Adults: Evidence from a Meta‐Analysis of Randomized Trials. J American Geriatrics Society. mai 2014;62(5):857‑64.

68.	Sardar P, Chatterjee S, Herzog E, Nairooz R, Mukherjee D, Halperin JL. Novel Oral Anticoagulants in Patients With Renal Insufficiency: A Meta-analysis of Randomized Trials. Canadian Journal of Cardiology. Elsevier BV; août 2014;30(8):888‑97.

69.	Sardar P, Chatterjee S, Mukherjee D. Efficacy and safety of new oral anticoagulants for extended treatment of venous thromboembolism: systematic review and meta-analyses of randomized controlled trials. Drugs. juill 2013;73(11):1171‑82.

70.	Sen P, Kundu A, Sardar P, Chatterjee S, Nairooz R, Amin H, et al. Outcomes After Cardioversion in Atrial Fibrillation Patients Treated with Non-Vitamin K Antagonist Oral Anticoagulants (NOACs): Insights from a Meta-Analysis. Am J Cardiovasc Drugs. févr 2016;16(1):33‑41.

71.	Senoo K, Kondo Y, Miyazawa K, Isogai T, Chun YH, Kobayashi Y. Safety and efficacy of direct oral anticoagulants over warfarin in Japanese patients with acute venous thromboembolism: A meta-analysis. Journal of Cardiology. Elsevier BV; mai 2017;69(5):763‑8.

72.	Sharma M, Cornelius VR, Patel JP, Davies JG, Molokhia M. Efficacy and Harms of Direct Oral Anticoagulants in the Elderly for Stroke Prevention in Atrial Fibrillation and Secondary Prevention of Venous Thromboembolism: Systematic Review and Meta-Analysis. Circulation. Ovid Technologies (Wolters Kluwer Health); 21 juill 2015;132(3):194‑204.

73.	Su Z, Zhang H, He W, Ma J, Zeng J, Jiang X. Meta-analysis of the efficacy and safety of non-vitamin K antagonist oral anticoagulants with warfarin in Latin American patients with atrial fibrillation. Medicine. Ovid Technologies (Wolters Kluwer Health); mai 2020;99(18):e19542.

74.	Sun MT, Wood MK, Chan W, Selva D, Sanders P, Casson RJ, et al. Risk of Intraocular Bleeding With Novel Oral Anticoagulants Compared With Warfarin: A Systematic Review and Meta-analysis. JAMA Ophthalmol. American Medical Association (AMA); 1 août 2017;135(8):864.

75.	Telles-Garcia N, Dahal K, Kocherla C, Lip GYH, Reddy P, Dominic P. Non-vitamin K antagonists oral anticoagulants are as safe and effective as warfarin for cardioversion of atrial fibrillation: A systematic review and meta-analysis. International Journal of Cardiology. Elsevier BV; oct 2018;268:143‑8.

76.	Thomopoulos C, Ntalakouras J, Polyzos D, Konstantinidis D, Palaiodimou L, Tsivgoulis G, et al. Net clinical benefit of a reduced dose of DOACs in non-valvular atrial fibrillation: A meta-analysis of randomized trials. Pharmacological Research. Elsevier BV; janv 2022;175:105902.

77.	Touma L, Filion KB, Atallah R, Eberg M, Eisenberg MJ. A Meta-Analysis of Randomized Controlled Trials of the Risk of Bleeding With Apixaban Versus Vitamin K Antagonists. The American Journal of Cardiology. Elsevier BV; févr 2015;115(4):533‑41.

78.	Uchino K. Dabigatran Association With Higher Risk of Acute Coronary Events: Meta-analysis of Noninferiority Randomized Controlled Trials. Arch Intern Med. American Medical Association (AMA); 12 mars 2012;172(5):397.

79.	Van Der Hulle T, Kooiman J, Den Exter PL, Dekkers OM, Klok FA, Huisman MV. Effectiveness and safety of novel oral anticoagulants as compared with vitamin K antagonists in the treatment of acute symptomatic venous thromboembolism: a systematic review and meta‐analysis. Journal of Thrombosis and Haemostasis. Elsevier BV; mars 2014;12(3):320‑8.

80.	Van Es N, Coppens M, Schulman S, Middeldorp S, Büller HR. Direct oral anticoagulants compared with vitamin K antagonists for acute venous thromboembolism: evidence from phase 3 trials. Blood. 18 sept 2014;124(12):1968‑75.

81.	Wang KL, Lopes RD, Patel MR, Büller HR, Tan DSY, Chiang CE, et al. Efficacy and safety of reduced-dose non-vitamin K antagonist oral anticoagulants in patients with atrial fibrillation: a meta-analysis of randomized controlled trials. European Heart Journal. Oxford University Press (OUP); 14 mai 2019;40(19):1492‑500.

82.	Wang M, Xie J, Zheng H, Wang S, Zhou R, Li W, et al. Wound complications and bleeding with new oral anticoagulants in patients undergoing total joint arthroplasty: A systematic review and meta‐analysis of randomized controlled trials. Brit J Clinical Pharma. Wiley; févr 2022;88(2):500‑13.

83.	Wang MK, Baskaran G, Razeghi G, Ma R, Park LS, Tannu M, et al. Bleeding Risks With Non–Vitamin K Oral Anticoagulants Versus Single Antiplatelet Therapy: A Systematic Review and Meta-analysis of Randomized Trials. Ann Intern Med. mars 2025;178(3):360‑8.

84.	Wu T, Lv C, Wu L, Chen W, Lv M, Jiang S, et al. Risk of intracranial hemorrhage with direct oral anticoagulants: a systematic review and meta-analysis of randomized controlled trials. J Neurol. Springer Science and Business Media LLC; févr 2022;269(2):664‑75.

85.	Xie J, Jiang M, Lin Y, Deng H, Xie X, Li L. Rivaroxaban versus Aspirin in Prevention of Venous Thromboembolism: A Meta-Analysis of 9 Randomized Controlled Trials comprising 7,656 Patients. Thromb Haemost. Georg Thieme Verlag KG; sept 2019;119(09):1517‑26.

86.	Xiong Q, Lau YC, Senoo K, Lane DA, Hong K, Lip GYH. Non‐vitamin K antagonist oral anticoagulants (NOACs) in patients with concomitant atrial fibrillation and heart failure: a systemic review and meta‐analysis of randomized trials. European J of Heart Fail. Wiley; nov 2015;17(11):1192‑200.

87.	Xiu-min L, Shi-guang S, Wei-dong Z. Apixaban versus enoxaparin for thromboprophylaxis after total hip or knee arthroplasty: a meta-analysis of randomized controlled trials. Chinese Medical Journal.

88.	Yoshida RDA, Yoshida WB, Maffei FHDA, El Dib R, Nunes R, Rollo HA. Systematic Review of Randomized Controlled Trials of New Anticoagulants for Venous Thromboembolism Prophylaxis in Major Orthopedic Surgeries, Compared With Enoxaparin. Annals of Vascular Surgery. avr 2013;27(3):355‑69.

89.	Yu Y bo, Liu J, Fu G hua, Fang R yuan, Gao F, Chu H min. Comparison of dabigatran and warfarin used in patients with non-valvular atrial fibrillation: Meta-analysis of random control trial. Medicine. Ovid Technologies (Wolters Kluwer Health); nov 2018;97(46):e12841.

90.	Yu Z, Shan P, Yang X, Lou X jiang. Comparison of efficiency and safety of rivaroxaban, apixaban and enoxaparin for thromboprophylaxis after arthroplastic surgery: a meta-analysis. Bioscience Reports. Portland Press Ltd.; 21 déc 2018;38(6).

91.	Zelniker TA, Ruff CT, Antman EM, Giugliano RP. The efficacy and safety of non-vitamin K antagonist oral anticoagulants in patients with atrial fibrillation and coronary artery disease: A meta-analysis of randomized trials. European Heart Journal: Acute Cardiovascular Care. Oxford University Press (OUP); sept 2019;8(6):554‑61.

92.	Zhao Y, Lu Y, Qin Y. A meta-analysis of randomized controlled trials of uninterrupted periprocedural anticoagulation strategy in patients undergoing atrial fibrillation catheter ablation. International Journal of Cardiology. Elsevier BV; nov 2018;270:167‑71.

93.	Zoppellaro G, Marchese GM, Squizzato A, Denas G, Patti G, De Caterina R, et al. Benefit of dual antithrombotic therapy with direct oral anticoagulants in patients with atrial fibrillation undergoing percutaneous coronary intervention: a systematic review and metaanalysis of randomized clinical trials. Intern Emerg Med. Springer Science and Business Media LLC; sept 2020;15(6):1093‑104.

| APPENDIX C  Checklist PRISMA 2020 | | | |
| --- | --- | --- | --- |
| **Section and Topic** | **Item #** | **Checklist item** | **Location where item is reported** |
| **TITLE** | | |  |
| Title | 1 | Identify the report as a systematic review. | Page 1 |
| **ABSTRACT** | | |  |
| Abstract | 2 | See the PRISMA 2020 for Abstracts checklist. | Page 2 |
| **INTRODUCTION** | | |  |
| Rationale | 3 | Describe the rationale for the review in the context of existing knowledge. | Page 4 |
| Objectives | 4 | Provide an explicit statement of the objective(s) or question(s) the review addresses. | Page 5, paragraph 1 |
| **METHODS** | | |  |
| Eligibility criteria | 5 | Specify the inclusion and exclusion criteria for the review and how studies were grouped for the syntheses. | Methods, page 5, paragraph 3, 4 |
| Information sources | 6 | Specify all databases, registers, websites, organisations, reference lists and other sources searched or consulted to identify studies. Specify the date when each source was last searched or consulted. | Methods, page 5, paragraph 2, |
| Search strategy | 7 | Present the full search strategies for all databases, registers and websites, including any filters and limits used. | Methods, page 5, paragraph 5 ; page 6, paragraphe 1 ; Appendix Table S1 |
| Selection process | 8 | Specify the methods used to decide whether a study met the inclusion criteria of the review, including how many reviewers screened each record and each report retrieved, whether they worked independently, and if applicable, details of automation tools used in the process. | Methods, page 6, paragraph 2 |
| Data collection process | 9 | Specify the methods used to collect data from reports, including how many reviewers collected data from each report, whether they worked independently, any processes for obtaining or confirming data from study investigators, and if applicable, details of automation tools used in the process. | Methods, page 6, paragraph 3 |
| Data items | 10a | List and define all outcomes for which data were sought. Specify whether all results that were compatible with each outcome domain in each study were sought (e.g. for all measures, time points, analyses), and if not, the methods used to decide which results to collect. | Methods, page 6, paragraph 3 |
|  | 10b | List and define all other variables for which data were sought (e.g. participant and intervention characteristics, funding sources). Describe any assumptions made about any missing or unclear information. | Methods, page 6, paragraph 3 |
| Study risk of bias assessment | 11 | Specify the methods used to assess risk of bias in the included studies, including details of the tool(s) used, how many reviewers assessed each study and whether they worked independently, and if applicable, details of automation tools used in the process. | Methods, page 7, paragraph 1 |
| Effect measures | 12 | Specify for each outcome the effect measure(s) (e.g. risk ratio, mean difference) used in the synthesis or presentation of results. | Methods, Page 7, paragraph 2 |
| Synthesis methods | 13a | Describe the processes used to decide which studies were eligible for each synthesis (e.g. tabulating the study intervention characteristics and comparing against the planned groups for each synthesis (item #5)). | Not relevant |
|  | 13b | Describe any methods required to prepare the data for presentation or synthesis, such as handling of missing summary statistics, or data conversions. | Not relevant |
|  | 13c | Describe any methods used to tabulate or visually display results of individual studies and syntheses. | Not relevant |
|  | 13d | Describe any methods used to synthesize results and provide a rationale for the choice(s). If meta-analysis was performed, describe the model(s), method(s) to identify the presence and extent of statistical heterogeneity, and software package(s) used. | Not relevant |
|  | 13e | Describe any methods used to explore possible causes of heterogeneity among study results (e.g. subgroup analysis, meta-regression). | Not relevant |
|  | 13f | Describe any sensitivity analyses conducted to assess robustness of the synthesized results. | Not relevant |
| Reporting bias assessment | 14 | Describe any methods used to assess risk of bias due to missing results in a synthesis (arising from reporting biases). | Not relevant |
| Certainty assessment | 15 | Describe any methods used to assess certainty (or confidence) in the body of evidence for an outcome. | Methods, Page 7, paragraph 4 |
| **RESULTS** | | |  |
| Study selection | 16a | Describe the results of the search and selection process, from the number of records identified in the search to the number of studies included in the review, ideally using a flow diagram. | Results, page 7, paragraph 5, 6 |
|  | 16b | Cite studies that might appear to meet the inclusion criteria, but which were excluded, and explain why they were excluded. | Figure 1, page 22; |
| Study characteristics | 17 | Cite each included study and present its characteristics. | Results, page 7, the last two paragraphs  Table 1, |
| Risk of bias in studies | 18 | Present assessments of risk of bias for each included study. | Results, page 7, last paragraph |
| Results of individual studies | 19 | For all outcomes, present, for each study: (a) summary statistics for each group (where appropriate) and (b) an effect estimate and its precision (e.g. confidence/credible interval), ideally using structured tables or plots. | Results, page 7, 8, 9, Table 2, 3, Figure 2 |
| Results of syntheses | 20a | For each synthesis, briefly summarise the characteristics and risk of bias among contributing studies. | Not relevant  (Results) |
|  | 20b | Present results of all statistical syntheses conducted. If meta-analysis was done, present for each the summary estimate and its precision (e.g. confidence/credible interval) and measures of statistical heterogeneity. If comparing groups, describe the direction of the effect. | Page 8, paragraph 3,4 |
|  | 20c | Present results of all investigations of possible causes of heterogeneity among study results. | Not relevant  (Results) |
|  | 20d | Present results of all sensitivity analyses conducted to assess the robustness of the synthesized results. | Not relevant  (Results) |
| Reporting biases | 21 | Present assessments of risk of bias due to missing results (arising from reporting biases) for each synthesis assessed. | Not relevant  (Results) |
| Certainty of evidence | 22 | Present assessments of certainty (or confidence) in the body of evidence for each outcome assessed. | Not relevant  (Results) |
| **DISCUSSION** | | |  |
| Discussion | 23a | Provide a general interpretation of the results in the context of other evidence. | Discussion, page 9, paragraph 3 |
|  | 23b | Discuss any limitations of the evidence included in the review. | Discussion, page 10, paragraph 4 |
|  | 23c | Discuss any limitations of the review processes used. | Discussion, page 10, paragraph 4 |
|  | 23d | Discuss implications of the results for practice, policy, and future research. | Discussion, page 10, paragraph 3 |
| **OTHER INFORMATION** | | |  |
| Registration and protocol | 24a | Provide registration information for the review, including register name and registration number, or state that the review was not registered. | Not relevent |
|  | 24b | Indicate where the review protocol can be accessed, or state that a protocol was not prepared. | Discussion, page 10, paragraph 4 |
|  | 24c | Describe and explain any amendments to information provided at registration or in the protocol. | No amendments |
| Support | 25 | Describe sources of financial or non-financial support for the review, and the role of the funders or sponsors in the review. | Page 12 |
| Competing interests | 26 | Declare any competing interests of review authors. | Page 12 |
| Availability of data, code and other materials | 27 | Report which of the following are publicly available and where they can be found: template data collection forms; data extracted from included studies; data used for all analyses; analytic code; any other materials used in the review. | Contact the authors, page 1 |
